# Evaluating CO_2_ breakthrough in a shaly caprock material: a multi-scale experimental approach

**DOI:** 10.1038/s41598-022-14793-8

**Published:** 2022-06-23

**Authors:** Eleni Stavropoulou, Lyesse Laloui

**Affiliations:** Laboratory for Soil Mechanics (LMS), EPFL-ENAC-LMS, Ecole Polytechnique Fédérale de Lausanne (EPFL), Station 18, 1015 Lausanne, Switzerland

**Keywords:** Carbon capture and storage, Geology, Geophysics, Characterization and analytical techniques, Environmental impact, Civil engineering

## Abstract

The potential of underground CO_2_ storage relies on the sealing efficiency of an overlaying caprock that acts as a geological barrier. Shales are considered as potential caprock formations thanks to their favourable hydro-mechanical properties. In this work the sealing capacity of Opalinus Clay shale to CO_2_ injection is studied by means of capillary entry-pressure and volumetric response. The overall objective of this work is to contribute to the safe design of a CO_2_ injection strategy by providing a better understanding of the geomechanical response of the caprock material to CO_2_ injection and eventual breakthrough at different scales. This is achieved by relating lab-measured hydro-mechanical properties of the studying caprock material (porosity, permeability, volumetric response) to field-related parameters (effective stress, injection pressure). A number of CO_2_ breakthrough tests is performed in Opalinus Clay samples under two different scales, meso and micro. At the meso-scale, CO_2_ injection is performed in oedometric conditions under different levels of axial effective stress in both gaseous or liquid phase. In parallel, the material’s transport properties in terms of water permeability are assessed before CO_2_ injection at each corresponding level of effective stress. The impact of CO_2_ phase and open porosity on the material’s CO_2_ entry pressure are demonstrated. The correlation between measured entry pressure and absolute permeability is discussed. A second testing campaign at a smaller scale is presented where CO_2_ breakthrough is for the first time identified with in-situ X-ray tomography. CO_2_ injection is performed under isotropic conditions on an Opalinus Clay micro-sample (micro-scale), and CO_2_ breakthrough is identified through quantitative image analysis based on the measured localised volumetric response of the material. This innovative methodology provides important insight into the anisotropic response of this complex material that is indispensable for its representative modelling in the context of safe geological CO_2_ storage.

## Introduction

Carbon Capture and Storage (CCS) is a technology that has been introduced by the petrol and gas industry since more than twenty years. However, large scale $\hbox {CO}_2$ storage sites have been limited around the world, with facilities mainly in the USA and northern Europe, until a few years ago when the momentum of CCS started growing back^1,2^. This increasing interest in CCS investment has been driven by the increasing need to reduce emissions as an effort to mitigate climate change while maintaining the energy demand. Since a couple of years, more countries around the globe are initiating $\hbox {CO}_2$ storage facilities that will enable energy transition while keeping $\hbox {CO}_2$ emissions low^3^.

**Figure 1:**
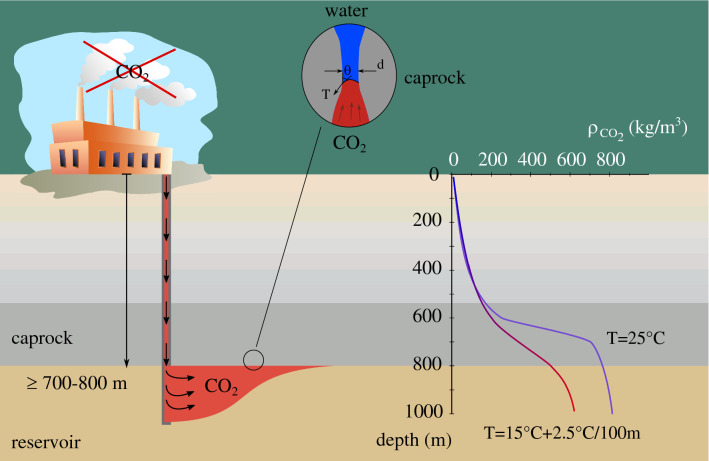
Schematic representation of underground $\hbox {CO}_2$ injection accompanied by the evolution of the $\hbox {CO}_2$ density with depth (considering hydrostatic pressure conditions): for a constant temperature of 25 $^{\circ }\hbox {C}$ and a thermal gradient of 2.5 $^{\circ }\hbox {C}$/100 m for a surface temperature equal to 15 $^{\circ }\hbox {C}$.

The technology of underground $\hbox {CO}_2$ storage is based on the same principles that nature has been employing to keep oil and gas underground over millions of years. Geological or structural trapping of $\hbox {CO}_2$ is the primary mechanism that prevents $\hbox {CO}_2$ migration from the storage reservoir to the surface and relies on the existence of an impermeable caprock formation that acts as a hydromechanical barrier. The $\hbox {CO}_2$ injection pressure in the reservoir is compensated by the capillary forces that are created in the caprock’s pore space and prevent $\hbox {CO}_2$ penetration and leakage to the surface. To achieve a higher storage volume, $\hbox {CO}_2$ is compressed and injected in the reservoir in a liquid (pressure, p $\ge$ 7.4 MPa) or supercritical form (pressure, p $\ge$ 7.4 MPa and temperature, T $\ge$ 31.2$^{\circ }\hbox {C}$). For this reason a minimum depth of 700 to 800 m is desired assuming hydrostatic conditions. Figure 1 illustrates the main principles of geological $\hbox {CO}_2$ storage together with the corresponding density evolution of $\hbox {CO}_2$ with depth, both for a constant temperature of 25 $^{\circ }\hbox {C}$ (testing conditions of this study) and assuming a thermal gradient of 2.5 $^{\circ }\hbox {C}$ per 100 m depth (surface temperature equal to 15 $^{\circ }\hbox {C}$) as suggested by Ref.^4^. Compressed $\hbox {CO}_2$ is usually injected in a brine saturated reservoir (usually sandstone or limestone) and because the density of the injected $\hbox {CO}_2$ is lower than that of the in-situ brine, $\hbox {CO}_2$ migrates upwards until in contact with the caprock; this is referred to as plume effect^5,6^ (see Fig. 1) or as formation dry-out^7,8^.

The minimum pressure of $\hbox {CO}_2$—or a non-wetting fluid more generally—that is required to displace the pore water of the caprock material is called entry pressure (PE) and it is defined as the difference between the $\hbox {CO}_2$ injection pressure ($\hbox {u}_{\text {CO}_2}$) and the pore water pressure ($\hbox {u}_{\text {w}}$): PE = $\hbox {u}_{\text {CO}_2}$ - $\hbox {u}_{\text {w}}$. The $\hbox {CO}_2$ entry pressure is also referred to as $\hbox {CO}_2$ *overpressure* or *threshold* pressure and in this work the concept explained by Ref.^9^ is considered for its measurement. Upon $\hbox {CO}_2$ injection, water is first displaced from the largest pores of a water saturated sample. Even though breakthrough may have not yet occured at this stage, i.e. the invading non-wetting fluid has not yet reached the other side of the sample due to local heterogeneities or due to the scale of observation, breakthrough and entry pressures are considered to be equal. From an experimental point of view the $\hbox {CO}_2$ injection pressure can be further increased in order to measure a breakthrough pressure that corresponds to the capillary pressure of the largest interconnected pores across the rock volume and is defined as:

1 $$\begin{aligned} \text {p}_{\text {c}} = \dfrac{4 \ \gamma \ \text {cos}\theta }{d}, \end{aligned}$$

where $\gamma$ (N/m) and $\theta$ (${}^{\circ }$) are the interfacial tension and contact angle of the wetting (water) / non-wetting ($\hbox {CO}_2$) interface respectively, and *d* (m) is the corresponding pore or throat diameter. This definition of capillary pressure describes the case of a single throat (see Fig. 1), it is thus somewhat simplistic for the description of capillarity of a natural heterogeneous material. Nevertheless, capillarity (and therefore breakthrough) is driven by the larger pores or throats of the material. In the case of an existing (micro-) fracture, this will be the principle flow channel and therefore, the definition of the entry pressure can be brought down to a single diameter; the minimum local fracture aperture.

It is obvious that capillarity is related to the shape and size of the porous space. A potential caprock material is expected to develop high capillary forces, therefore very small pore sizes are required. Shales (or other mudrocks) are usually identified as preferable caprock formations thanks to their favourable properties among which except for high capillarity are the very low permeability, self-sealing and high sorption capacity. Shales have indeed a very small dominant pore size with ranges that vary from a couple of nm to a couple of hundreds of nm^10^. It has been shown that porosity decreases exponentially as depth increases in normally-compacted formations^11^, and consequently it is commonly accepted that porosity decreases exponentially with effective stress^12^. Depth (overburden stress) and effective stress are parameters related to site conditions and have to be taken into account for the characterisation of a caprock material. The impact of these site parameters on the material’s hydromechanical properties are reflected through porosity.

Previous studies have investigated the entry pressure of shaly caprock materials upon $\hbox {CO}_2$ injection^13,14^ and they have identified an entry pressure that varies from a couple of MPa up to close to 18 MPa. These results are encouraging and demonstrate the material’s sealing potential, however, they have been obtained based on different techniques with limited emphasis on the applied hydromechanical boundary conditions which represent the different possible storage or injection conditions. The lack of experimental results under known geomechanical boundary conditions motivated the current study, the objective of which is to relate the sealing response of the material with site related parameters (effective stress and $\hbox {CO}_2$ injection pressure) and transport properties (water permeability and $\hbox {CO}_2$ capillary entry pressure) that can be assessed from lab-scale characterisation.

## Tested material

**Figure 2:**
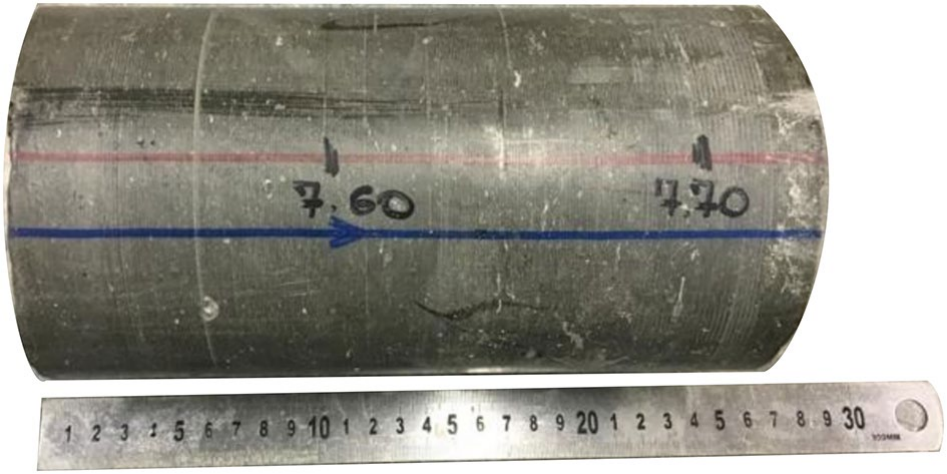
Opalinus Clay core sample.

An Opalinus Clay (OPA) core extracted from borehole D5S (corresponding to a total overburden of 262.7 m) in the Mont Terri Underground Research Laboratory (URL) in Switzerland has been used for the experimental activities of this work. The OPA core is shown in Fig. 2 and it is mainly composed of clay particles, i.e. extracted from the shaly facies of Opalinus Clay stratum^17^.

The basic properties of the intact material (“as-received”) have been identified in the lab. More precisely, the water content of the core is measured equal to 6.47% based on mass measurement before and after oven drying at 105 $^{\circ }\hbox {C}$. The solid grain density is measured equal to $\rho _{\text {s}}$ = 2.73 g/cm$^3$ according to the water pycnometer technique on crushed material that was sieved at 0.5 mm. Finally, the bulk density is measured on a cylindrical sample from caliper measurements of the height and diameter and is found equal to $\rho = 2.37$ g/cm$^3$. From these parameters, the initial void ratio and and the degree of saturation are calculated equal to e =   0.226 and $\hbox {S}_r$ = 78% respectively.

**Figure 3:**
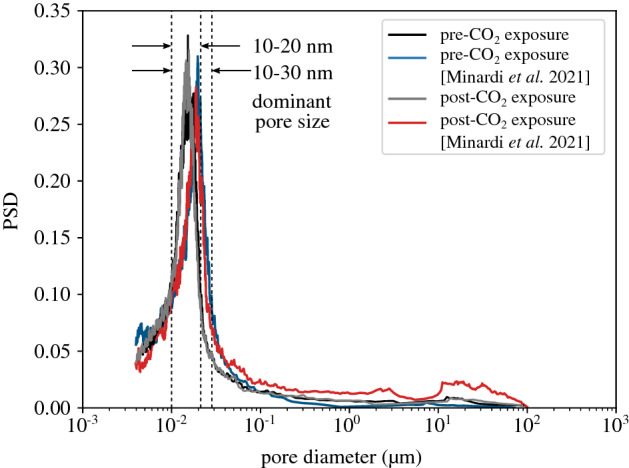
Pore size distribution (PSD) of the tested material, before and after exposure to $\hbox {CO}_2$.

The pore size distribution (PSD) of the Opalinus Clay core is measured based on the Mercury Intrusion Porosimetry (MIP) technique and it is presented in Fig. 3 together with the results reported in Ref.^14^. The clay-rich material presents in both cases a single dominant pore size; uni-fractional distribution. The slight difference between the range of the two shaly cores (10–20 nm from current study vs. 10–30 nm from ref.^14^) could be attributed to the accuracy and limitations of the MIP technique^15^ or it can be additionally related to the mineralogical composition of the studied cores (see Table 1); a higher clay content and lower carbonate and quartz content measured in the core of the present study and therefore more intraparticle to interparticle porosity^16^.

In the same figure, the PSD of the material after $\hbox {CO}_2$ breakthrough is presented. Similarly to the Ref.^14^ observations, no significant differences are visible on the dominant pore size before and after exposure to $\hbox {CO}_2$. In addition, the obtained PSD is not affected in the higher range between 10 $\upmu$m and 100 $\upmu$m unlike the results reported by^14^. This may be related to the duration of $\hbox {CO}_2$ exposure; while in this study each $\hbox {CO}_2$ injection lasts an average of one week and it is followed by water resaturation, Ref.^14^ exposed the Opalinus Clay sample to $\hbox {CO}_2$ for 105 days. This long-term exposure may have partially dried the sample and created fissures that manifest in the higher range of the PSD. Nevertheless, it should be reminded that the accuracy of the MIP technique in shales presents several limitations related to the detection of macro-pores due to the presence of fissures and surface effects^18^.

**Table 1:** Mineralogical composition of the Opalinus Clay; current study and from Ref.^14^.

Shaly OPA (wt%)	Quartz	Carbonate	Clay	Other
OPA core from current study	9	15	74	2
Minardi et al.^41^	6	20	68	6

## Methodology and experimental campaign

Breakthrough is commonly identified in low permeable media based on the stepwise technique^9^. $\hbox {CO}_2$ is placed in contact with the upstream surface of a water-saturated sample at an initial pressure equal to the pore water pressure of the material. The $\hbox {CO}_2$ pressure is then increased in constant pressure steps, the resolution and duration of which depend on the sample’s permeability and the required accuracy for the capillary entry pressure definition. When the $\hbox {CO}_2$ overpressure ($\hbox {CO}_2$ pressure minus pore water pressure) is higher than the capillary entry pressure of the material, water is displaced out of the sample and when flow increase is observed on the other side of the sample breakthrough is identified. This means that once breakthrough has occurred, the $\hbox {CO}_2$ entry pressure of the material is defined within the range of the last $\hbox {CO}_2$ pressure step. In this campaign, $\hbox {CO}_2$ breakthrough experiments in Opalinus Clay have been performed under two different scales and boundary conditions. All tests have taken place under controlled temperature conditions equal to 25 $^{\circ }\hbox {C}$ ± 0.5 $^{\circ }\hbox {C}$ and the description of each testing procedure is presented in the following.

### Meso-scale oedometric breakthrough tests

**Figure 4:**
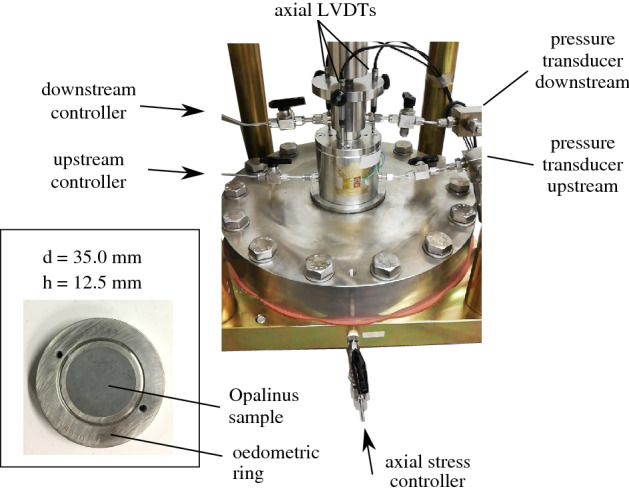
High pressure oedometric cell and Opalinus Clay specimen in the oedometric ring.

A first series of $\hbox {CO}_2$ injections under oedometric conditions has been performed on two cylindrical OPA samples (samples OPA-1 and OPA-2) with dimensions h = 35.0 mm and d = 12.5 mm as shown in Fig. 4. A high pressure oedometric device located in the Laboratory of Soil Mechanics in EPFL has been employed for the tests, where the axial load (max. 100 MPa) and the upstream and downstream pore pressures (max. 16 MPa each) can be independently applied and monitored both in pressure and volume^19^. The axial displacement is measured by three LVDTs while an individual pressure transducer is connected upstream and downstream close to the cell for additional validation of the applied pressures.

**Figure 5:**
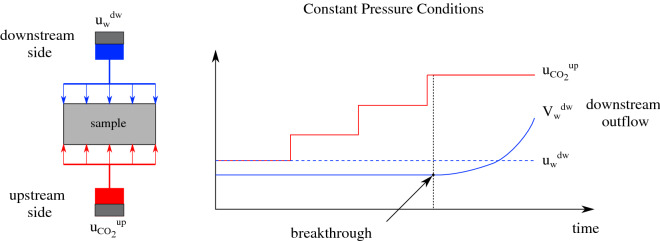
$\hbox {CO}_2$ breakthrough testing layout under constant pressure conditions.

$\hbox {CO}_2$ is injected at the upstream side of the sample at increasing steps of 1 MPa (constant pressure steps) while applying constant pressure conditions at the downstream reservoir, as illustrated in Fig. 5. Once a sudden increase at the downstream outflow volume is observed, breakthrough is identified and the $\hbox {CO}_2$ entry pressure of the material is defined as PE = $\hbox {u}_{\text {CO}_2, \text {up}}$ − $\hbox {u}_{\text {w,dw}}$. Evidently, the precision of the breakthrough and consequently entry pressure is directly related to the resolution of the applied steps, i.e. 1 MPa. $\hbox {CO}_2$ injection under constant pore pressure conditions downstream is considered more representative to in-situ conditions since in a simple case of a homogeneous caprock without for example low permeability faults, the conditions are closer to drained (constant pressure). From an experimental point of view, constant volume conditions downstream (undrained conditions) result in immediate pressure increase downstream due to water’s incompressibility.

**Figure 6:**
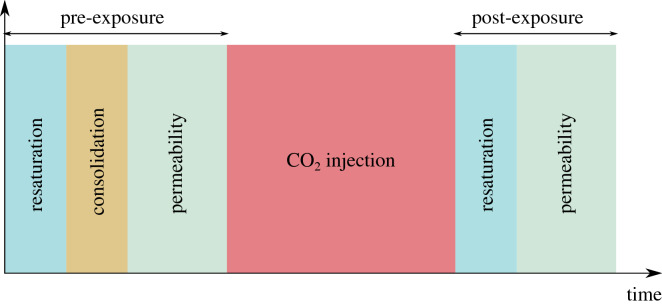
General timeline of each breakthrough test.

Each breakthrough test lasts an average of 15 days, during which a series of different phases takes place in the order illustrated in Fig. 6. First, the sample is saturated with water (initial saturation under constant volume conditions for the assessment of the swelling pressure of the sample) after which a total axial load is applied. After consolidation is completed (average 2 days) the water permeability of the material is assessed (1–3 days).

The pre-exposure phase is then followed by the $\hbox {CO}_2$ injection phase which can last up to 8–10 days; average injection duration per step $\approx$ 2 days. Finally, after breakthrough has occured, the sample is resaturated with water and post-injection permeability is in most cases measured. The different breakthrough tests have been performed on two Opalinus Clay samples from the same core; sample 1 and sample 2. Both samples are cut parallel to the bedding with a mechanical lathe and placed in the oedometric ring immediately after unsealing the core. Sample 2 is tested immediately after mounted in the oedometric ring while sample 1 is sealed in a vacuumed neoprane envelope and stored in controlled humidity (85%) and atmospheric pressure conditions.

### Micro-scale isotropic compression breakthrough tests

In addition to the oedometric conditions, $\hbox {CO}_2$ breakthrough tests under isotropic compression conditions have been performed using a “home-made” polyetheretherketone (PEEK) cell, the so called *PEEKcell* that has been developed by the authors. PEEKcell allows testing of small cylindrical samples (5 mm $\times$ 5 mm) during live X-ray tomography and it is designed to sustain 20 MPa and 40 $^{\circ }\hbox {C}$ maximum pressure and temperature respectively. The consideration of such small size samples aims first of all to achieve reduced time durations of the different hydromechanical phenomena—consolidation (based on Terzaghi’s consolidation theory) and entry pressure/breakthrough—in this very low permeable material (improved temporal resolution), but also to the increase of spatial resolution of the acquired X-ray scans.

**Figure 7:**
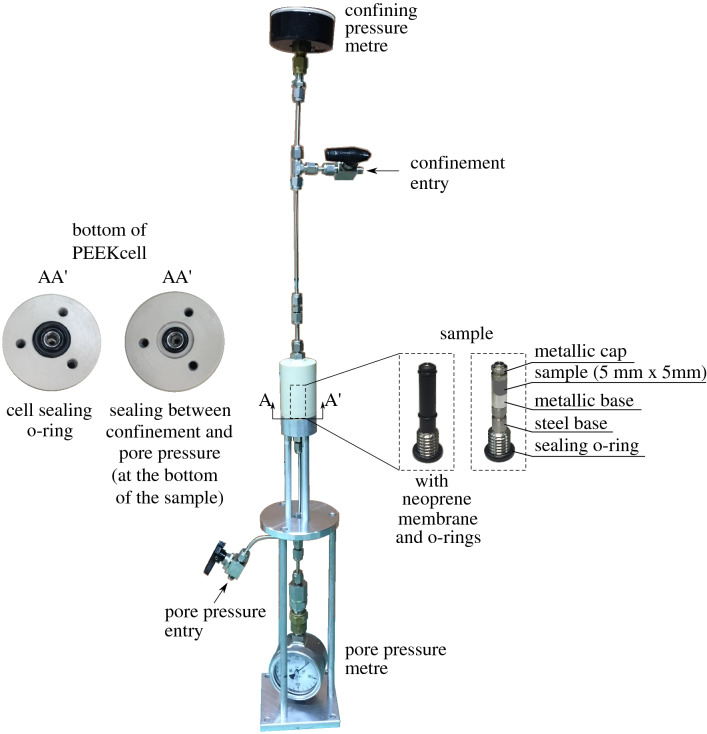
PEEKcell—confinement cell for live $\hbox {CO}_2$ injection in the X-ray tomograph.

The setup that has been developed and employed during this study is presented in Fig. 7. The sample is placed between an aluminium base and cap and it is sealed on a steel base with a neoprene membrane and two o-rings (top and bottom). The confining pressure is applied from an entry at the top of the cell while the pore pressure is accessed from the bottom of the cell directly to the sample (upstream). The tests are performed under constant volume conditions, i.e. once the target confining stress and pore pressure are applied, the lines (valves) are closed and any potential leakage is monitored from the pressure metres that are connected in between.

**Figure 8:**
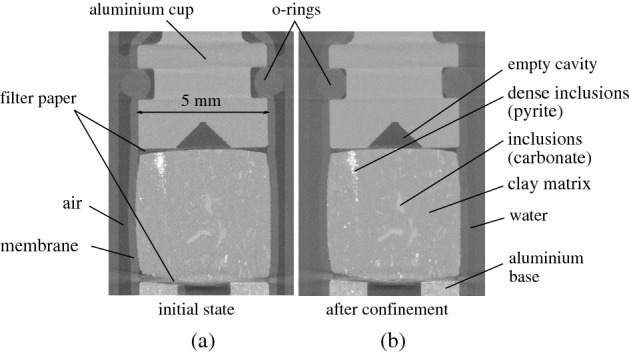
Sample setup in the PEEKcell from two vertical slices (**a**) before and (**b**) after confinement.

Given the fact that there is no downstream pressure control, the pore water pressure of the sample is null and therefore PE = $\hbox {u}_{\text {CO}_2}$. In order to avoid the undrained constant volume conditions that were discussed in the case of the oedometer, a cavity of an approximate volume equal to the average pore volume of the sample is created on the top cap of the sample. In this way, breakthrough can physically occur by displacing the pore water in the existing empty space. The described configuration of this top part of the sample setup is shown in Fig. 8 from two vertical slices of X-ray images before and after the application of confinement (spatial resolution 7.85 $\upmu$m/px).

**Figure 9:**
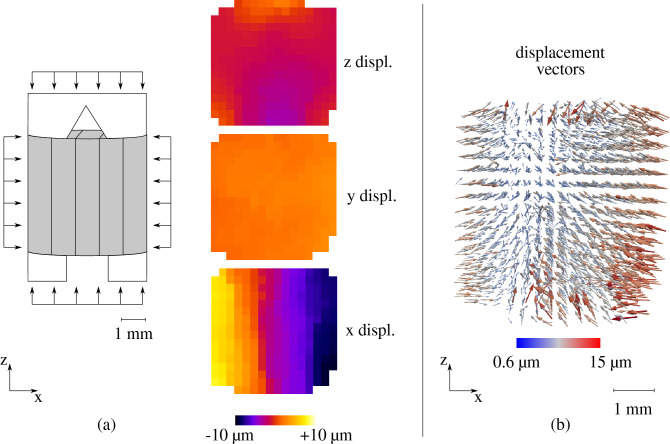
Application of 10 MPa isotropic confinement with PEEKcell: (**a**) sample sketch (vertical bedding) and calculated displacement maps (vertical middle slice), (**b**) calculated displacement vectors—the size and colour of the vectors represent the absolute displacement value, while displacement direction is indicated from the arrows’ direction.

As revealed from the setup’s cross-section in Fig. 8, the top and bottom sides of the sample are not entirely in contact with the cap and base. This may have an impact on the distribution of the applied confinement. To evaluate this potential drawback, the displacement maps of the sample along the three axes are calculated for an applied confinement of 10 MPa. As shown in Fig. 9a, a localised displacement of a couple of microns is measured along the vertical axis (z), at the top and bottom of the sample. Along the two horizontal axes (x and y) a homogeneously distributed displacement is obtained, which is as expected more pronounced along the axis x, i.e. perpendicular to the bedding orientation of the sample. The error of the measurement has been evaluated by comparing two consecutive identical X-ray scans at the initial state of the sample, i.e. under undonfined conditions. After calculation of the displacement field between these two “identical” scans, the error has been measured equal to 0.04 px, i.e. 0.3 $\upmu$m, however, this is an overestimation since the sample has already experienced some strain from one scan to the other (possibly due to minor drying). For a better understanding of the local displacement within the sample, the local displacement vectors are plotted in Fig. 9b. An overall isotropic compaction is obtained, proving the successful application of confinement, with only some slightly more pronounced displacement activity at the upper and lower edges of the sample. In any case, in the context of breakthrough testing, confinement perpendicular to the sample’s bedding is what is of utmost importance to ensure closure of potentially pre-existing micro-fissures.

Similarly to the larger scale breakthrough tests $\hbox {CO}_2$ injection is performed according to the stepwise technique, this time in constant pressure steps equal to 2 MPa. The tested sample is initially resaturated under controlled humidity and atmospheric pressure conditions until mass stabilisation is reached. Then the sample is mounted inside PEEKcell and confinement is applied. Thanks to the small size of the sample, consolidation is completed shortly after the application of the confining pressure ($\approx$ 1 h) and finally, $\hbox {CO}_2$ is injected at increasing pressure levels. The mechanical response of the sample is evaluated with image analysis on the different acquired scans before and after each loading phase.

## Oedometric breakthrough testing results

In this section, the results obtained from the different breakthrough tests under oedometric conditions are presented. First, the $\hbox {CO}_2$ entry pressure of the material is assessed under different levels of axial effective stress and different $\hbox {CO}_2$ phases (gaseous and liquid). Then the hydraulic behaviour of the material is investigated based on the acquired water permeability at different levels of applied effective stress in order to investigate its association to the corresponding measured entry pressure. The axial effective stress, $\sigma _{\text {ax}}'$, is considered based on the Terzaghi’s equation, i.e., $\sigma _{\text {ax}}' = \sigma _{\text {ax}}$ − $\ \text {u}_{\text {p}}$ where, $\sigma _{\text {ax}}$ is the total axial compressive stress and $\ \text {u}_{\text {p}}$ is the pore-fluid pressure. It is important to point out the given definition refers to the effective stress applied on the sample before $\hbox {CO}_2$ breakthrough, i.e. while the material is fully saturated with water. After $\hbox {CO}_2$ breakthrough, $\hbox {CO}_2$ injection is stopped and the sample is resaturated with water for the measurement of the corresponding permeability (single phase flow).

### Assessment of the $\hbox {CO}_2$ entry-pressure

In this campaign, a series of breakthrough tests has been performed under constant pressure boundary conditions at the downstream water reservoir. In the following, the results of four breakthrough tests (performed on sample OPA-2) are explained in detail and presented in chronological order: loading history can have an irreversible impact on the structural (porosity) and therefore hydromechanical properties of the material. During each subsequent breakthrough test the same or an increased level of axial effective stress is applied. In the following results, $\hbox {CO}_2$ is injected under two levels of axial effective stress (10 MPa and 22 MPa) and two levels of pore water pressure are considered (2 MPa and 8 MPa). The initial $\hbox {CO}_2$ injection pressure is equal to the given pore water pressure corresponding to injection of both gaseous ($\hbox {u}_{\text {CO}_2, {\text {init}}} =$ 2 MPa) and liquid $\hbox {CO}_2$ ($\hbox {u}_{\text {CO}_2, {\text {init}}} =$ 8 MPa).

**Figure 10:**
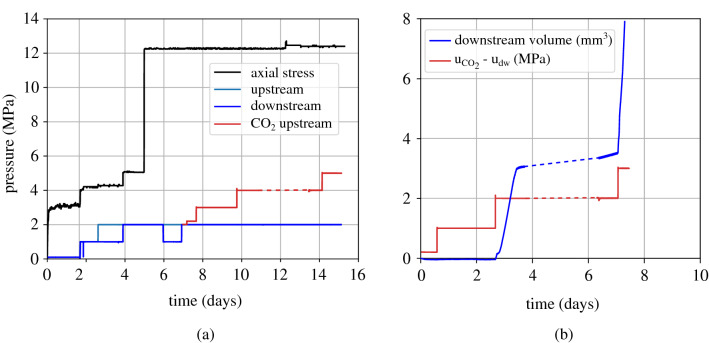
Breakthrough test with *gaseous* $\hbox {CO}_2$ injection under an initial axial effective stress = 10 MPa, (**a**) applied loading: axial stress, upstream and downstream pressures, (**b**) $\hbox {CO}_2$ overpressure $\hbox {u}_{\text {CO}_2} - \text {u}_{\text {w,dw}}$ and corresponding water volume downstream.

First, the sample is saturated under low pore water pressure ($\hbox {u}_{\text {dw}}$ = 200 kPa) and constant volume conditions, i.e. zero axial displacement, in order to assess the swelling pressure which is stabilised to 3.2 MPa (see Fig. 10a t = 2 days). Afterwards, the total axial stress and the pore water pressure, both upstream and downstream, are increased by 1 MPa in order to further enhance saturation and upon stabilisation of the axial displacement one day after, a constant pore pressure gradient of 1 MPa between the upstream and downstream sides of the sample is applied for the measurement of water permeability, once steady state conditions are achieved (t = 2 to 4 days). The total axial stress is then increased to a target value equal to 12 MPa and upon the end of consolidation, i.e. stabilisation of the axial displacement (t = 5 days), the water permeability is again evaluated. This is the end of the pre-exposure phase.

As shown in Fig. 10a, $\hbox {CO}_2$ is introduced at the upstream side of the sample at a pressure equal to the downstream water pressure i.e. 2 MPa. Figure 10b shows the evolution of the downstream water volume outflow during $\hbox {CO}_2$ injection (blue plot) together with the corresponding $\hbox {CO}_2$ overpressure $\hbox {u}_{\text {CO}_2}$ - $\text {u}_{\text {w,dw}}$ (red plot). Upon overpressure increase from 1 to 2 MPa, a sudden increase of the water outflow at the downstream side is obtained indicating the excess of capillary forces in the pores of the material. $\hbox {CO}_2$ pressure is further increased (after the dashed lines in Fig. 10) to ensure that breakthrough has occured and as expected the downstream outflow is further increased.

**Figure 11:**
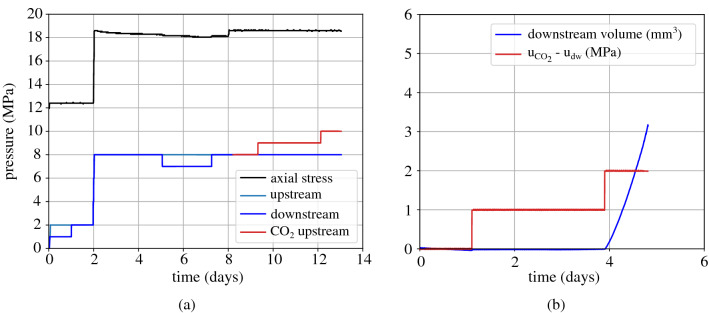
Breakthrough test with *liquid* $\hbox {CO}_2$ injection under an initial axial effective stress = 10 MPa, (**a**) applied loading: axial stress, upstream and downstream pressures, (**b**) $\hbox {CO}_2$ overpressure $\hbox {u}_{\text {CO}_2} - \text {u}_{\text {w,dw}}$ and corresponding water volume downstream.

Figure 11 shows the results of the proceeding breakthrough test that is performed under the same axial effective stress (i.e. 10 MPa) and liquid $\hbox {CO}_2$ injection; pore water pressure and initial $\hbox {CO}_2$ injection pressure equal to 8 MPa. Given the chronological order of the presentation of the tests, the first 2 days of the graph in Fig. 11a shows the water resaturation of the sample after the first gaseous $\hbox {CO}_2$ injection. Then the total axial load is increased to 18.2 MPa followed by a consolidation phase that lasted around 3 days. The sample’s water permeability is then assessed with the application of a pressure gradient equal to 1 MPa during 2 days when steady state conditions have been achieved. Finally, the two pore pressures upstream and downstream are set to a pressure equal to 8 MPa before the beginning of injection. Figure 11b shows the evolution of the $\hbox {CO}_2$ overpressure with time, toghether with the downstream water volume outflow. A clear increase of the water outflow is obtained upon $\hbox {CO}_2$ overpressure increase from 1 to 2 MPa.

**Figure 12:**
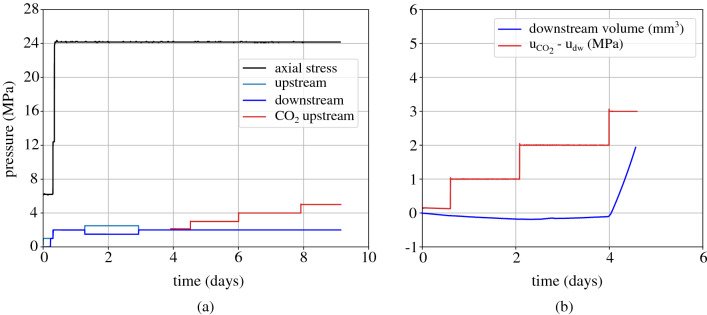
Breakthrough test with *gaseous* $\hbox {CO}_2$ injection under an initial axial effective stress = 22 MPa, (**a**) applied loading: axial stress, upstream and downstream pressures, (**b**) $\hbox {CO}_2$ overpressure ${\hbox {u}}_{\text {CO}_2} - {\text {u}}_{\text {w,dw}}$ and corresponding water volume downstream.

After the end of the second breakthrough test ($\sigma _{\text {ax}} = 18.2$ MPa and $\hbox {u}_{\text {dw,w}} = 8$ MPa) the sample is unloaded to a total axial stress equal to 6 MPa and resaturated with water under low pore pressure equal to 1 MPa. After 1 day, the total axial load and pore water pressure (at both sides) are increased to 24 MPa and 2 MPa respectively as shown in Fig. 12a. Upon completion of consolidation, the water permeability of the sample under 22 MPa axial effective stress is measured with the application of a pressure gradient equal to 1 MPa. The two sides of the sample are then set back to a pressure equal to 2 MPa and after equilibration, gaseous $\hbox {CO}_2$ injection from the upstream side is initiated under the same pore pressure, i.e. 2 MPa. The upstream $\hbox {CO}_2$ pressure is then increased with 1 MPa steps that last an average time equal to 1.5–2 days (Fig. 12b). In this test, breakthrough is identified during $\hbox {CO}_2$ overpressure increase from 2 to 3 MPa as shown from the downstream water outflow on the downstream side in Fig. 12b.

**Figure 13:**
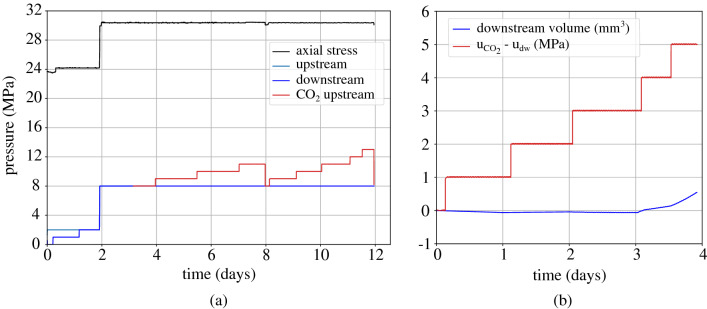
Breakthrough test with *liquid* $\hbox {CO}_2$ injection under an initial axial effective stress = 22 MPa, (**a**) applied loading: axial stress, upstream and downstream pressures, (**b**) $\hbox {CO}_2$ overpressure $\hbox {u}_{\text {CO}_2} - \text {u}_{\text {w,dw}}$ and corresponding water volume downstream.

A last breakthrough test is presented in this section under the same axial effective stress (22 MPa) and liquid $\hbox {CO}_2$ injection. As shown in Fig. 13a after resaturation the sample’s total axial load and pore water pressure are increased to 30 MPa and 8 MPa. Liquid $\hbox {CO}_2$ is then injected from the upstream side at an initial pressure equal to 8 MPa. Step-wise injection has been re-initiated at 1 MPa pressure steps that have been halted due to leak issues on the downstream side. A second step-wise injection follows and it is presented in terms of $\hbox {CO}_2$ overpressure and downstream water outflow in Fig. 13. An increase of the water outflow is observed upon increase of the $\hbox {CO}_2$ overpressure from 3 to 4 MPa, while an additional $\hbox {CO}_2$ pressure increase has been performed to ensure breakthrough; indeed, the downstream water outflow increases even more.

The results of the presented breakthrough tests reveal a higher $\hbox {CO}_2$ entry pressure under higher axial effective stress. Under low effective stress (here 10 MPa) the impact of the $\hbox {CO}_2$ phase does not directly reflect on the measured entry pressure—obviously under the given resolution of $\hbox {CO}_2$ pressure increase, i.e. 1 MPa. Under a higher and more realistic level of effective (22 MPa) the $\hbox {CO}_2$ entry pressure is higher when $\hbox {CO}_2$ is injected in liquid form. These results together with those of additional liquid $\hbox {CO}_2$ injection tests are assembled and discussed in “Entry-pressure evolution with applied effective stress” section.

### Hydraulic response

Before further analysis and interpertation of the breakthrough results, the hydraulic response of the material in terms of water permeability is presented and discussed. It has been demonstrated by various experimental results on multiple shale samples that the level of the applied effective stress has an impact on the permeability of shales, in particular at levels higher than the pre-consolidation stress of the material^20–23^. Some researchers reported a higher stress sensitivity of permeability for samples with high clay and low carbonate content^24,25^. The decrease of permeability is mainly attributed to a decrease in connected pore volume network with the increase of the effective stress, in particular those pores which act as critical links in the network^26^.

Different empirical equations have been proposed in the literature for the description of acquired experimental data, most commonly in the form of exponential functions^27–29^ or power laws^30,31^. Even though Ref.^32^ showed that the power law permeability models can be approximated by exponential equations, in this study we chose a power law model to describe the relation between effective stress and measured water permeability, k (m$^2$), of our shaly samples:

2 $$\begin{aligned} \text {k} = \text {k}_0 \Big (\dfrac{\sigma _{ax}'}{\sigma _{ax,0}'}\Big )^\alpha , \end{aligned}$$

where $\hbox {k}_0$ (m$^2$) is the permeability that corresponds to an effective stress $\sigma _{\text {ax,0}}'$ (MPa) and $\alpha$ is a material constant.

The water permeability of the two Opalinus samples has been measured under different levels of axial effective stress (oedometric conditions) applying a constant head flow^33,34^. A water pressure difference equal to 1 MPa is applied between the upstream and downstream side of the sample and the permeability k (m$^2$) of the medium is calculated through the hydraulic conductivity K (m/s) while considering the fluid’s dynamic viscosity $\eta _f$ (Pa$\cdot$s), density $\rho _f$ (kg/m$^3$) and the gravity acceleration g (m/s$^2$):

3 $$\begin{aligned} \text {k} = \text {K} \ \dfrac{\eta _f}{\rho _f \ \text {g}}. \end{aligned}$$

Based on the Darcy’s law, the hydraulic conductivity can be calculated as follows:

4 $$\begin{aligned} \text {K} = \text {q}_f \ \dfrac{\rho _f \ \text {g} \ \text {L}}{A \ \Delta \text {P}}, \end{aligned}$$

where, $\hbox {q}_f$ (m$^3$/s) is the volumetric flow, L (m) the height of the sample, A (m$^2$) the area of the sample, $\Delta \hbox {P}_f$ (Pa) the applied pressure difference.

**Figure 14:**
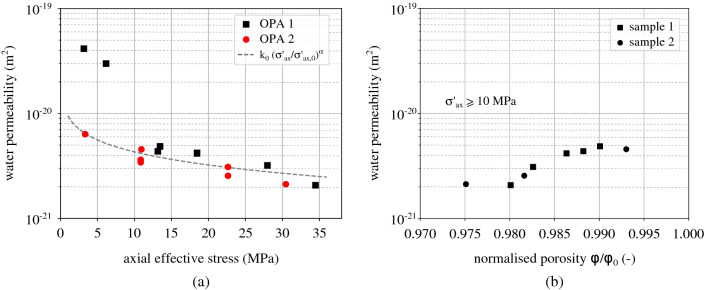
(**a**) Water permeability and corresponding applied axial effective stress—the fitted line does not consider the measured permeability under axial effective stress lower than 7 MPa for sample OPA-1 (two square points), (**b**) evolution of water permeability with the corresponding normalised porosity $\phi /\phi _0$ for $\sigma _{\text {ax}}'\ge$ 10 MPa (sample 1: $\phi _0$= 16.03% and sample 2: $\phi _0$= 18.43%).

Figure 14a shows the values of water permeability that have been measured for each sample under the corresponding applied axial effective stress. As expected, permeability decreases with the increase of the applied effective stress. The results of the two samples are compatible for applied levels of effective stress higher than 10 MPa, while under lower effective stress a large variability is observed. This response can be explained by the pre-existence of micro-cracks that under low confinement remain open and dominate the flow. Upon load increase, pre-existing fissures close and the flow is again controlled by the porosity of the sample. It is indeed most likely that dessication fissures have been created in sample OPA-1 which was tested one month after the opening of the Opalinus Clay core. For this reason the fitting of equation Eq. (2) does not consider the two first points of OPA-1 (square points). The fitted parameters are $\hbox {k}_0$ = 2.15 $\times 10^{-19}$ m$^2$, $\sigma _{\text {ax,0}}'$ = 0.0004 MPa and $\alpha$ =  0.38. The Opalinus Clay (and shales in general) is an anisotropic material the hydromechanical response of which (including permeability) depend on the bedding orientation. In this work, the tested samples have bedding parallel to the flow (vertical bedding) and therefore anisotropy related effects on tortuosity for example are not considered.

The decreasing water permeability with increasing effective stress is due to the porosity decrease. The available porosity values with the corresponding water permeability under the same level of effective stress are plotted in Fig. 14b. The porosity $\phi$ of each sample is calculated based on the axial displacement evolution and while not all the displacement values are available, the plot properly demonstrates the correspondance between the two material properties; higher water permeability for higher levels of normalised porosity. The calculated $\phi$ values are normalised with the initial porosity of each sample $\phi _0$ under no effective stress. These are equal to 16.03% for sample 1 and 18.43% for sample 2.

### Entry-pressure evolution with applied effective stress

In this section, the level of effective stress will be evaluated in relation with the corresponding level of applied effective stress. As shown above, the hydraulic response of the studied material in terms of measured water permeability has been related to the applied levels of axial effective stress by means of evolving porosity. However, the link between porosity and entry pressure (PE) is less straight forward since the definition of the latter is a function of a pore/throat of diameter *d*:

5 $$\begin{aligned} \text {PE} = \text {p}_{\text {c}} = \dfrac{4 \ \gamma \ \text {cos}\theta }{d}. \end{aligned}$$

From this fundamental definition it can be deduced that breakthrough will occur at the pore/throat with the higher diameter. In shales, at low levels of effective stress, it is likely that pre-existing micro-fissures still remain open and hence breakthrough will be driven by them (largest *d*). As effective stress increases, these micro-fissures will tend to close and therefore both throat size and porosity will decrease. Reference^23^ reported both decreasing porosity and dominant pore size in shallow Opalinus Clay with depth. This similar tendency between the two parameters (pore size and porosity) and depth (overburden stress) remains empirical, nevertheless it serves as an extra motivation in this study for the evaluation of the impact of effective stress on the corresponding entry pressure value by means of pore structure modifications.

**Figure 15:**
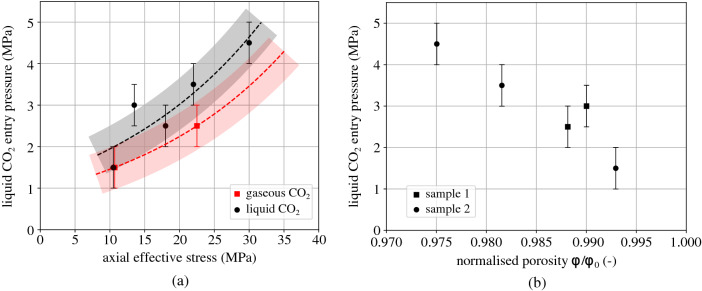
(**a**) Measured $\hbox {CO}_2$ entry pressure and corresponding applied axial effective stress, (**b**) measured $\hbox {CO}_2$ entry pressure for liquid $\hbox {CO}_2$ injection and corresponding normalised porosity $\phi /\phi _0$ (sample 1: $\phi _0$ = 16.03% and sample 2: $\phi _0$ = 18.43%).

The results of the measured $\hbox {CO}_2$ entry pressure of the tested Opalinus Clay with the corresponding axial effective stress are summarised in Table 2 and plotted in Fig. 15a. The effective stress is calculated considering the applied total axial stress and the downstream pore water pressure which corresponds to the initial applied $\hbox {CO}_2$ injection pressure. The results from the gaseous $\hbox {CO}_2$ injection are separated from the liquid $\hbox {CO}_2$ results, since surface tension ($\gamma$) and wetting properties (contact angle, $\theta$) of the $\hbox {CO}_2$/water interface change with pressure (and therefore phase)^35,36^. The obtained values of $\hbox {CO}_2$ entry pressure are consistent for liquid $\hbox {CO}_2$ injection (indicative values of $\gamma$ = 30 mN/m and $\theta =40^{\circ }$ from Ref.^36^), however, for gaseous $\hbox {CO}_2$ injection (lower $\hbox {CO}_2$ pressure) the equivalent values of surface angle and contact angle result in higher entry pressure. This discrepancy between experimental results and theory requires further investigation for the better understanding of the sealing response of the given material and its implication to real storage. For instance, the consideration of additional hydromechanical mechanisms related to phenomena such as partial desaturation and suction increase from gaseous $\hbox {CO}_2$ injection, may be necessary for a more appropriate interpretation of these results.

**Table 2:** Applied pressure conditions and measured $\hbox {CO}_2$ entry pressure from the different breakthrough tests on the two Opalinus Clay samples.

Sample	$\sigma _{\text {ax}}'$ (MPa)	u$_{\text {w,dw}}$ (MPa)	$\hbox {CO}_2$ phase	PE (MPa)
OPA 2	10.3	2.0	gas	$1.5 \pm 0.5$
	10.9	8.0	liq	$1.5 \pm 0.5$
	22.2	2.0	gas	$2.5 \pm 0.5$
	22.6	8.0	liq	$3.5 \pm 0.5$
	30.3	8.0	liq	4.5 ± 0.5
OPA 1	13.4	8.0	liq	$3.0 \pm 0.5$
	18.3	8.0	liq	2.5 ± 0.5

For both $\hbox {CO}_2$ phases the measured entry pressure increases with the increase of the axial effective stress. This result reveals the importance of the accurate definition of the hydromechanical boundary state of the caprock material for the evaluation of its entry pressure. Even though the obtained general trend shows a lower entry pressure upon gaseous $\hbox {CO}_2$ injection compared to the corresponding liquid, for low effective stress (10 MPa), a similar entry pressure has been measured for both $\hbox {CO}_2$ phases—within the 1 MPa range of the increasing injection step. This result reveals the high impact of open porosity (open fissures) to the sealing capacity of the material that dominates the response. As the effective stress increases, pre-existing cracks close and porosity decreases, thus the phase difference reflects in a more significant way on the response. The results are fitted using an exponential fit based on empirical laws of porosity evolution with effective stress^37,38^.

The available values of the calculated normalised porosity $\phi /\phi _0$ of the two samples are also plotted with the corresponding measured entry values (Fig. 15b); these are only for liquid $\hbox {CO}_2$ injection. Impressively enough, a near linear trend is obtained, highlighting the importance of open porosity on the sealing capacity of the material; higher $\hbox {CO}_2$ entry pressure for lower levels of normalised porosity. This is consistent with the definition of the capillary entry pressure, nevertheless, more experimental results are required for more concrete conclusions.

## Small-scale isotropic compression breakthrough testing

The results of the oedometric breakthrough tests confirmed that flow (water permeability) and sealing capacity ($\hbox {CO}_2$ entry pressure) of the studied caprock material are driven by the open porosity and potentially pre-existing micro-fissures. This outcome motivated a parallel campaign where $\hbox {CO}_2$ injection is performed in much smaller cylindrical Opalinus Clay samples ($5 \times 5$ mm) and the kinematics are analysed with *real-time* X-ray tomography. Live X-ray scanning allows the observation and quantification of localised structural changes, which in the case of shales is hard to be achieved at a pore scale (nanometric scale). Nevertheless, resolutions of a few microns can reveal existing micro-fissures and their evolution upon confinement and $\hbox {CO}_2$ injection. A spatial resolution of 7.85 $\upmu$m/px is achieved and each scan has lasted 30 min.

The Opalinus Clay sample has been resaturated progressively after exposure to atmospheric pressure and controlled relative humidity (RH) conditions. Two saturated saline solutions have been used, first a NaCl solution corresponding to RH = 75% followed by a $\hbox {K}_2\hbox {SO}_4$ corresponding to RH = 98%^39^ over a period of at least 10 days each at 25 $^{\circ }\hbox {C}$. A progressive exposure to higher RH has been preferred, given the high sensitivity of the material to water changes and the atmospheric pressure conditions during resaturation. The weight of the samples has been followed daily and upon stabilisation, a close-to-full saturation is considered, corresponding to a measured water content $\hbox {w}_{\text {resat}}$ = 6.67%, i.e. within the range of full saturation^40^. The sample is then mounted in PEEKcell the lines of which are vacuumed at low back pressure both upstream and downstream.

**Figure 16:**
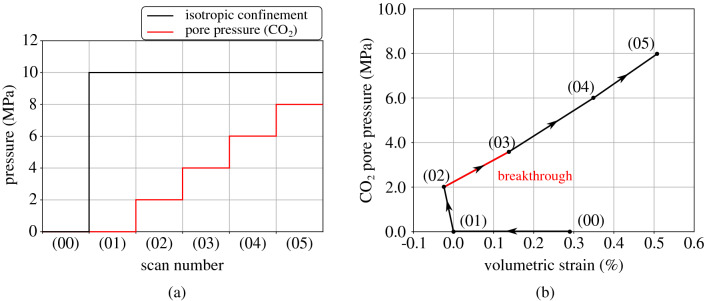
Breakthrough test under isotropic confinement, (**a**) applied pressure at each scan, (**b**) normalised total volumetric strain (reference scan: 01) with applied $\hbox {CO}_2$ pore pressure.

A first scan under unconfined conditions is taken after which isotropic confinement is applied. Based on the size of the sample, consolidation is expected to occur faster than a conventional size sample, i.e. consolidation is supposed to be completed in approximately one hour. After the end of the consolidation phase, $\hbox {CO}_2$ is injected into the sample from the upstream side at steps of 2 MPa. Six scans have been performed in total, as shown in Fig. 16a; (00): initial unconfined state, (01): after p = 10 MPa isotropic confinement, (02): $\hbox {CO}_2$ injection, $\hbox {u}_{{\hbox {CO}_2}}$ = 2 MPa, (03): $\hbox {CO}_2$ injection, $\hbox {u}_{{\hbox {CO}_2}}$ = 4 MPa, (04): $\hbox {CO}_2$ injection, $\hbox {u}_{{\hbox {CO}_2}}$ = 6 MPa, (05): $\hbox {CO}_2$ injection, $\hbox {u}_{{\hbox {CO}_2}}$ = 8 MPa.

The volumetric evolution of the sample has been measured directly from the acquired images by performing a total registration between each scan and scan (01), i.e. after isotropic confinement (p = 10 MPa and $\hbox {u}_{{\hbox {CO}_2}}$ = 0). All other acquired volumes have been normalised with respect to the reference scan and the evolution of the volumetric strain with the applied effective stress is plotted in Fig. 16b. As the graph shows, an initial compaction takes place after the application of confinement (00 $\rightarrow$ 01). The volumetric strain during the application of 10 MPa confinement is equal to − 0.29% and it is comparable to the volumetric strain of an equivalent oedometric test after application of axial total stress of 12 MPa (see Fig. 10a) that is measured equal to − 0.52%. In theory, higher volumetric strain is expected for the same effective pressure in oedometric conditions (effective axial stress) compared to isotropic (effective isotropic stress). The difference in the mechanical response between the two testing campaigns can be attributed to several factors including sample variability (damage during preparation, pre-existing fissures), displacement measurement error (lvdt resolution vs image analysis), full vs partial water saturation of the sample etc. $\hbox {CO}_2$ injection at 2 MPa does not affect the sample in any significant way, only a slight further compaction is observed (01 $\rightarrow$ 02). During the next $\hbox {CO}_2$ increase at 3.6 MPa (02 $\rightarrow$ 03) the sample starts swelling and the volumetric response continues to be dilatant upon further increase of the $\hbox {CO}_2$ pressure (03 $\rightarrow$ 05). This change in volumetric response denotes the occurrence of breakthrough throughout the sample and a corresponding entry pressure between 2 and 3.6 MPa. This result is consistent with the results from the bigger scale breakthrough test under a similar axial effective stress (2 MPa). The volumetric response of the sample after breakthrough (02 $\rightarrow$ 05) evolves in a rather linear way, indicating that for the given test conditions, breakthrough takes place within the elastic domain without causing irreversible strain e.g. crack opening. This has to be further confirmed in a future campaign with the application of an unloading step.

**Figure 17:**
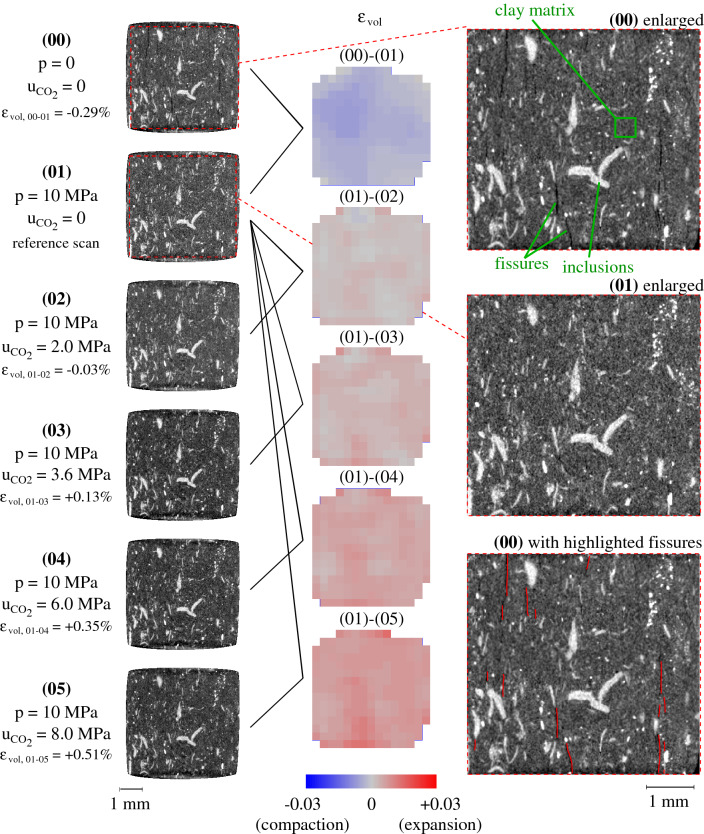
Middle vertical slices for each loading step and the resulting volumetric strain maps.

The middle vertical slice from each scan is presented in Fig. 17. As highlighted from the enlarged scans (00) and (01), unconfined state and after confinement respectively, even at this sample scale and scan resolution there are pre-existing micro-fissures. The open micro-fissures of scan (00) are highlighted in red in the same figure. These pre-existing micro-cracks disappear after the application of confinement and are no more detectable at the given resolution even after the application of the highest $\hbox {CO}_2$ injection pressure (8 MPa). Reference^41^ also observed sealing of hydraulic fractures from X-ray tomography imaging after increased confining stress. Even though fissures are not the sole contributor to inelastic response, the fact that do not reappear may further support an elastic mechanical response of the sample at the given test conditions. The vertical orientation of the micro-fissures confirms a similar bedding to the samples tested under oedometric conditions.

In addition to total volume changes, local Digital Voxel Correlation (DVC) has been performed in order to measure the localised strains within the sample^42^. All DVC have been performed using scan (01) as the reference 3D image: (00) $\rightarrow$ (01), (01) $\rightarrow$ (02), (01) $\rightarrow$ (03), (01) $\rightarrow$ (04) and (01) $\rightarrow$ (05). The volumetric strain maps are presented in the same Fig. 17 and as indicated from the colourbar below, the blue colour describes compaction, grey colour no volume changes and red colour expansion. Similarly to the global volumetric evolution, upon confinement an overall compaction is measured, and after the second step of $\hbox {CO}_2$ injection swelling starts and progressively increases. Localised swelling corresponds to local porosity changes which can serve as preferential $\hbox {CO}_2$ breakthrough pathways. When $\hbox {CO}_2$ breaks through the sample, the effective stress decreases locally and swelling occurs. It is interesting to note that these volumetric maps reveal a high swelling activity on locations where the pre-existing micro-fissures have been observed—this is particularly visible on the strain map of the last injection step. Even though reopening of the micro-fissures after $\hbox {CO}_2$ injection is not visible directly from the scans at this resolution, the localised volumetric expansion suggests their impact on the sealing capacity of the material. This is consistent with the interpretation of the previous oedometric results, where under low effective stress (under 10 MPa) variability of water permeability is impacted by the existence of micro-cracks. These results demonstrate the great potential of imaging tools for a better understanding of the hydro-mechanical response of the caprock material upon fluid injection or relevant processes.

**Figure 18:**
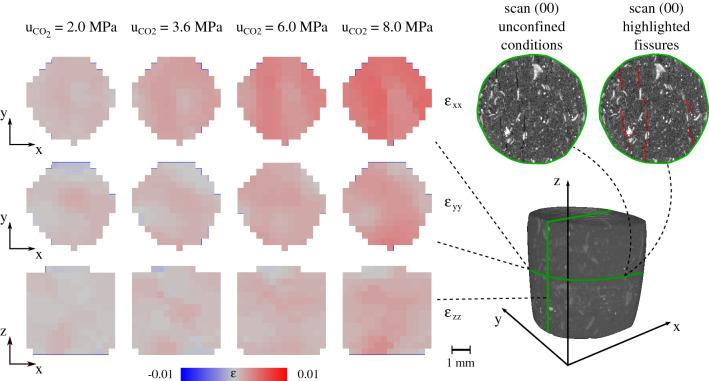
Directional strain maps of middle slices for each $\hbox {CO}_2$ injection pressure.

Finally, in order to explore the impact of bedding on the volumetric behaviour of the sample, the different strain components have been calculated for each injection step (Fig. 18). Deformation along the x axis ($\varepsilon _{\text {xx}}$), i.e. perpendicular to the bedding, is clearly more pronounced compared to the two other axes ($\varepsilon _{\text {yy}}$ and $\varepsilon _{\text {zz}}$), revealing the highly anisotropic response of the sample. More precisely, a higher $\varepsilon _{\text {xx}}$ strain activity is noticed at locations where pre-existing fissures have been identified before the application of confinement, as highlighted in the same figure. The localised strain along the other two axes also evolves in a non-isotropic way, indicating a local porosity evolution that is driven by both bedding and local breakdown of the effective stress due to $\hbox {CO}_2$ breakthrough.

## Discussion

The overall results from the breakthrough tests at both scales show the impact of applied effective stress on the measured $\hbox {CO}_2$ entry pressure by means of connected porosity. At the smaller scale, the sample dimensions are more representative of an elementary volume size, i.e. bulk matrix. Nevertheless, X-ray images revealed the existance of relaxation-related micro-fissures under unconfined conditions even at that micro-scale. At the given resolution, the contribution of these micro-cracks to the $\hbox {CO}_2$ breakthrough is not evident as they disappear upon application of confinement (10 MPa) and do not reappear even after $\hbox {CO}_2$ breakthrough. Nevertheless, 3D image analysis of the various X-ray images during $\hbox {CO}_2$ injection reveals a pronounced localised volumetric activity at locations that can be qualitatively related to locations of pre-existing cracks.

At the meso-scale tests (oedometric conditions), the impact of connected porosity is indirectly demonstrated by means of water permeability which decreases with the increase of the applied axial effective stress. For low levels of effective stress (under 10 MPa), any porosity evolution is dominated by the opening/closing of pre-existing fissures rather than pore collapsing, as demonstrated by the variable permeability between different samples. At higher levels of effective stress and in particular at levels past the in-situ pre-consolidation stress of the material (which has been reported in literature in a range of values between 15 and 18 MPa^23,43^), it is the evolution of actual matrix porosity that manifests and dominates the hydraulic and sealing response of the material.

**Figure 19:**
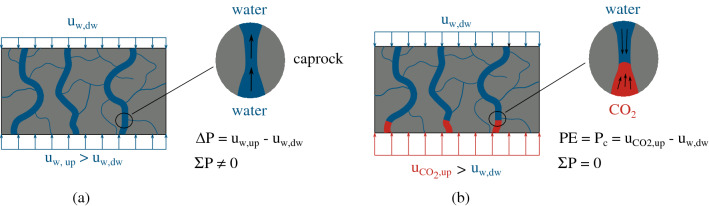
Water permeability and corresponding measured entry pressure.

Based on these meso-scale results, a correlation between entry pressure and absolute water permeability is investigated. Relating a hydromechanical property of the material (water permeability) with its sealing capacity upon $\hbox {CO}_2$ injection ($\hbox {CO}_2$ entry pressure) is an important asset for the design of an injection strategy. However, before further elaboration, it is important to distinguish the different states of the sample during the identification and measurement of these two different properties, a schematic representation of which is attempted in Fig. 19. On one hand, during the measurement of water permeability the sample is subjected to single fluid flow conditions (Fig. 19a), while on the other hand, entry pressure is defined during equilibrium conditions (Fig. 19b) where the water capillary pressure in the pores prohibits $\hbox {CO}_2$ flow in the sample. From a practical point of view, the definition of the entry pressure is achieved experimentally only upon flow observation ($\hbox {CO}_2$ penetration in the pore space) which at the same time signals the end of the test. Therefore, in the current study no multi-phase fluid flow or relative permeability are considered for the analysis and interepretation of the results, which in the authors’ opinion it is very advantageous since this approach involves a simpler experimental protocol.

**Figure 20:**
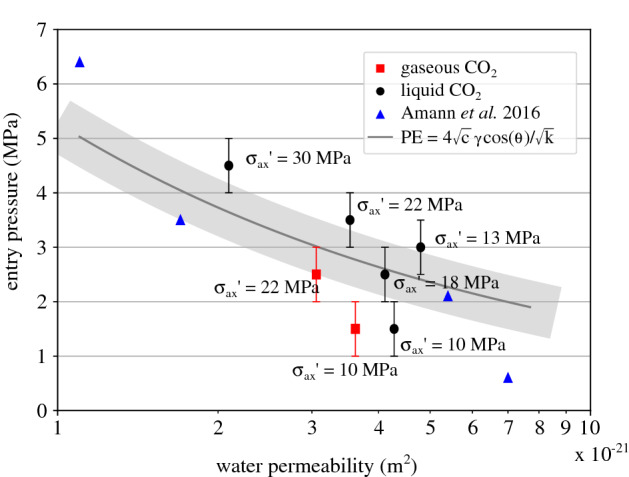
Water permeability and corresponding measured $\hbox {CO}_2$ entry pressure.

The experimental entry pressure results (both liquid and gaseous $\hbox {CO}_2$ injection) are plotted together with the corresponding water permeability of the material that has been measured under *the same effective stress levels* are presented in Fig. 20. Four additional measurements from Ref.^13^ are also included. There is a clear trend between the two plotted parameters; a higher $\hbox {CO}_2$ entry pressure corresponds to boundary conditions (applied effective stress) where water permeability is lower. The observed trend can be interpreted again through open porosity. Reference^44^ showed the correlation between permeability and a so called *estimated pore size* $\hbox {d}_{{sur}}$ (m):

6 $$\begin{aligned} \text {k} = \text {c} \cdot \text {d}_{sur}^2, \end{aligned}$$

where c is a scalar and $\hbox {d}_{{sur}}$ is a function of porosity ($\phi$—m$^3$/m$^3$), specific surface area ($\hbox {S}_s$—m$^2$/g) and mass density ($\rho$—g/cm$^3$). Their results are in line with the Kozeny–Carman equation^45,46^:

7 $$\begin{aligned} \text {k} = \dfrac{\phi }{32 \tau ^2} \ \text {d}_{sur}^2, \end{aligned}$$

where $\tau$ (m/m) is the tortuosity. In the current study, a single bedding orientation is considered (vertical, i.e. parallel to the injection), therefore tortuosity is considered to be a constant between the different samples. To this end, and based on the rationale (see “Entry-pressure evolution with applied effective stress” section) that relates the impact of effective stress to both porosity and pore size, the entry pressure can be related to the material’s permeability from Eqs. (5) and (6) through the consideration of an *estimated pore size*:

8 $$\begin{aligned} \text {PE} = \dfrac{4\sqrt{c} \ \gamma \ \text {cos} \theta }{\sqrt{k}}. \end{aligned}$$

From Eqs. (5) and (6) the $\hbox {CO}_2$ entry pressure can be related to the corresponding water permeability as described by Eq. (8). The different measured results of water permeability and entry pressure at corresponding levels of effective stress are plotted in Fig. 20. The two sets of parameters are fitted based on Eq. (8) considering a 1 MPa error. A mor significant scattering of the points that correspond to lower levels of effective stress is observed, once again due to higher variability of active/open porosity. Additionaly, a different line should be fitted for liquid or gaseous $\hbox {CO}_2$ injection, however, due to the limited number of results a single fit is considered. This last remark points out the need of more experimental results under known stress conditions for the calibration and improvement of this relation; experimental results that cannot be easily found in the existing literature for shales.

## Conclusions and perspectives

The presented work aims to provide an insight in the sealing response of the Opalinus Clay by means of entry pressure levels upon $\hbox {CO}_2$ injection while emphasizing on the importance of the applied hydro-mechanical conditions. A series of stepwise breakthrough tests have been performed under oedometric conditions. Gaseous and liquid $\hbox {CO}_2$ has been injected from the upstream side of the sample and constant pore water pressure conditions have been maintained at the downstream reservoir. Breakthrough has been investigated under different levels of applied axial effective stress in order to evaluate the impact of connected porosity on the sealing capacity of the material. Overall, higher entry pressure has been measured for higher levels of axial effective stress. For a given effective stress the entry pressure is higher for liquid $\hbox {CO}_2$ injection rather than gaseous. Under low levels of effective stress the impact of the injected $\hbox {CO}_2$ phase was not as significant (within the 1 MPa measurement error); this response has been attributed to the existence of open micro-cracks that dominate the response compared to the $\hbox {CO}_2$ phase. When higher loading is applied, open porosity decreases and pre-existing micro-fissures close, therefore the sealing capacity of the material improves.

The entry-pressure dependence on the porosity evolution motivated the investigation of the material’s water permeability; a material property that is also affected by the active porosity—and therefore applied levels of effective stress—and which is relatively easily assessed by standard laboratory hydromechanical testing. The measured water permeability of two Opalinus Clay samples has been assesed; lower permeability has been measured for higher levels of applied effective stress. Under lower loading, a variability on the obtained results between the two samples is observed which then decreases and the flow response becomes more consistent for higher confinement. The obtained variability can again be attributed to the existence of open micro-cracks under low effective stress that dominate the flow.

To further confirm the interpretation of the hydromechanical results, an original methodology has been proposed and demonstrated where $\hbox {CO}_2$ breakthrough is studied during real-time X-ray tomography. $\hbox {CO}_2$ is injected in a caprock micro-sample under isotropically confined conditions using a new experimental mini-device, so called PEEKcell, that is designed for live X-ray tomography. The kinematics of the sample at different levels of hydromechanical loading and $\hbox {CO}_2$ injection pressure have been analysed directly from the acquired 3D images. Under unconfined conditions, the X-ray scans revealed the existence of micro-fissures (resolution 7.85 $\upmu$m/px) in the visually intact 5 mm × 5 mm cylindrical sample. Upon confinement, these micro-fissures close and they are not anymore visible even after $\hbox {CO}_2$ breakthrough. $\hbox {CO}_2$ breakthrough is for the first time identified from the volumetric response of the caprock sample through quantitative image analysis of X-ray tomography images. The localised kinematics have shown highly anisotropic response of the sample and have suggested the impact of pre-existing fissures on the sealing capacity of the material. These results show the high potential of this methodology for a more accurate understanding of the non-homogeneous volumetric response of caprocks upon $\hbox {CO}_2$ breakthrough while embracing the material’s heterogeneity. This is particularly important for the development of representative models that can safely predict the material’s sealing capacity based on its micro-structural properties. Of particular interest for future studies remains the impact of pre-existing micro-fissures, with more precise (and quantitative) analysis of the preferential $\hbox {CO}_2$ flow network.

The sealing capacity of Opalinus Clay as a potential caprock formation has been investigated under different loading conditions and the response has been related to the material’s water permeability by means of active porosity. This approach is very promising and could be adopted for the design of a site selection or $\hbox {CO}_2$ injection strategy where depth and site conditions can be related to the corresponding hydromechanical boundary conditions while the caprock’s sealing capacity can be predicted by means of corresponding water permeability. For the assessment of a more robust hydromechanical model, more experimental results under defined stress conditions are required. Additionally, supercritical $\hbox {CO}_2$ injection should be performed in the future to further represent real site conditions.

## Data Availability

The raw X-ray datasets and the results of digital volume correlation are available on Zenodo, 10.5281/zenodo.5939022. The remaining used and/or analysed data during the current study are available from the corresponding author on reasonable request.

## References

[1] IEA (2020). CCUS in Clean Energy Transitions.

[2] IEA (2019). The Role of CO2 Storage.

[3] IEA (2021). CCUS Around the World.

[4] Metz B, Davidson O, De Coninck HC, Loos M, Meyer L (2005). IPCC Special Report on Carbon Dioxide Capture and Storage.

[5] Doughty C (2010). Investigation of CO2 plume behavior for a large-scale pilot test of geologic carbon storage in a saline formation. Transp. Porous Media.

[6] Espinoza DN, Santamarina JC (2017). CO2 breakthrough-Caprock sealing efficiency and integrity for carbon geological storage. Int. J. Greenhouse Gas Control.

[7] Pruess K, Müller N (2009). Formation dry-out from CO2 injection into saline aquifers: 1. Effects of solids precipitation and their mitigation. Water Resour. Res..

[8] Lima MG, Javanmard H, Vogler D, Saar MO, Kong XZ (2021). Flow-through drying during CO2 injection into brine-filled natural fractures: A tale of effective normal stress. Int. J. Greenhouse Gas Control.

[9] Boulin PF, Bretonnier P, Vassil V, Samouillet A, Fleury M, Lombard JM (2013). Sealing efficiency of caprocks: Experimental investigation of entry pressure measurement methods. Mar. Pet. Geol..

[10] Marschall P, Horseman S, Gimmi T (2005). Characterisation of gas transport properties of the Opalinus Clay, a potential host rock formation for radioactive waste disposal. Oil Gas Sci. Technol..

[11] Mondol NH, Bjørlykke K, Jahren J, Høeg K (2007). Experimental mechanical compaction of clay mineral aggregates—Changes in physical properties of mudstones during burial. Mar. Pet. Geol..

[12] Flemings PB, Stump BB, Finkbeiner T, Zoback M (2002). Flow focusing in overpressured sandstones: Theory, observations, and applications. Am. J. Sci..

[13] Amann-Hildenbrand A, Krooss BM, Bertier P, Busch A (2015). Laboratory testing procedure for CO2 capillary entry pressures on caprocks. Carbon Dioxide Capt. Storage Deep Geol. Format..

[14] Minardi A, Stavropoulou E, Kim T, Ferrari A, Laloui L (2021). Experimental assessment of the hydro-mechanical behaviour of a shale caprock during CO2 injection. Int. J. Greenhouse Gas Control.

[15] Comisky, J. T., Santiago, M., McCollom, B., Buddhala, A., & Newsham, K. E. Sample size effects on the application of mercury injection capillary pressure for determining the storage capacity of tight gas and oil shales. *In Canadian Unconventional Resources Conference. OnePetro* (2011).

[16] Klaver J, Desbois G, Littke R, Urai JL (2015). BIB-SEM characterization of pore space morphology and distribution in postmature to overmature samples from the Haynesville and Bossier Shales. Mar. Pet. Geol..

[17] Bossart, P., & Thury, M. *Mont Terri Rock Laboratory. Project, Programme 1996 to 2007 and Results. Wabern: Reports of the Swiss Geological Survey. Mont Terri Project*, 3 (2008).

[18] Minardi A (2018). Hydro-mechanical Characterization of Gas Shales and Opalinus Clay Shale in Partially Saturated Conditions.

[19] Ferrari A, Favero V, Laloui L (2016). One-dimensional compression and consolidation of shales. Int. J. Rock Mech. Min. Sci..

[20] Katsube TJ, Williamson MA (1994). Effects of diagenesis on shale nano-pore structure and implications for sealing capacity. Clay Miner..

[21] Bustin, R. M., Bustin, A. M., Cui, A., Ross, D., & Pathi, V. M. Impact of shale properties on pore structure and storage characteristics. In *SPE Shale Gas Production Conference. OnePetro* (2008).

[22] Bhandari AR, Flemings PB, Polito PJ, Cronin MB, Bryant SL (2015). Anisotropy and stress dependence of permeability in the Barnett shale. Transp. Porous Media.

[23] Crisci E, Ferrari A, Giger SB, Laloui L (2019). Hydro-mechanical behaviour of shallow Opalinus Clay shale. Eng. Geol..

[24] Ghanizadeh A, Gasparik M, Amann-Hildenbrand A, Gensterblum Y, Krooss BM (2014). Experimental study of fluid transport processes in the matrix system of the European organic-rich shales: I. Scandinavian Alum Shale. Mar. Pet. Geol..

[25] Fink R, Krooss BM, Amann-Hildenbrand A (2017). Stress-dependence of porosity and permeability of the Upper Jurassic Bossier shale: An experimental study. Geol. Soc. Lond. Spl. Publ..

[26] Kwon O, Kronenberg AK, Gangi AF, Johnson B, Herbert BE (2004). Permeability of illite-bearing shale: 1. Anisotropy and effects of clay content and loading. J. Geophys. Res. Solid Earth.

[27] Brace W, Walsh JB, Frangos WT (1968). Permeability of granite under high pressure. J. Geophys. Res..

[28] Chalmers GR, Ross DJ, Bustin RM (2012). Geological controls on matrix permeability of Devonian Gas Shales in the Horn River and Liard basins, northeastern British Columbia, Canada. Int. J. Coal Geol..

[29] Cui G, Liu J, Wei M, Shi R, Elsworth D (2018). Why shale permeability changes under variable effective stresses: New insights. Fuel.

[30] Katsube TJ, Mudford BS, Best ME (1991). Petrophysical characteristics of shales from the Scotian shelf. Geophysics.

[31] Ghabezloo S, Sulem J, Guédon S, Martineau F (2009). Effective stress law for the permeability of a limestone. Int. J. Rock Mech. Min. Sci..

[32] Shi JQ, Durucan S (2016). Near-exponential relationship between effective stress and permeability of porous rocks revealed in Gangi’s phenomenological models and application to gas shales. Int. J. Coal Geol..

[33] Darcy H (1856). Les Fontaines Publiques de la ville de Dijon: Exposition et Application.

[34] Renard P, Genty A, Stauffer F (2001). Laboratory determination of the full permeability tensor. J. Geophys. Res. Solid Earth.

[35] Wang S, Edwards IM, Clarens AF (2013). Wettability phenomena at the CO2-brine-mineral interface: Implications for geologic carbon sequestration. Environ. Sci. Technol..

[36] Sarmadivaleh M, Al-Yaseri AZ, Iglauer S (2015). Influence of temperature and pressure on quartz-water-CO2 contact angle and CO2-water interfacial tension. J. Colloid Interface Sci..

[37] Tsuji T, Tokuyama H, Costa Pisani P, Moore G (2008). Effective stress and pore pressure in the Nankai accretionary prism off the Muroto Peninsula, southwestern Japan. J. Geophys. Res. Solid Earth.

[38] Zhang J (2013). Effective stress, porosity, velocity and abnormal pore pressure prediction accounting for compaction disequilibrium and unloading. Mar. Pet. Geol..

[39] Romero, E. (2001). Controlled-suction techniques. *4o Simpósio Brasileiro de Solos Nâo Saturados* (eds. Gehling & Schnaid, F.), 535–542.

[40] Bossart P (2011). Characteristics of the Opalinus Clay at Mont Terri.

[41] Voltolini M, Ajo-Franklin JB (2020). The sealing mechanisms of a fracture in opalinus clay as revealed by in situ synchrotron X-ray micro-tomography. Front. Earth Sci..

[42] Stamati O, Asnd E, Roubin E, Cailletaud R, Wiebicke M, Pinzon G, Couture C, Hurley R, Caulk R, Caillerie D, Matsushima T, Birmpilis G (2020). Spam: Software for practical analysis of materials. J. Open Source Softw..

[43] Giger SB, Ewy RT, Favero V, Stankovic R, Keller LM (2018). Consolidated-undrained triaxial testing of Opalinus Clay: Results and method validation. Geomech. Energy Environ..

[44] Cardona A, Santamarina JC (2020). Carbonate rocks: Matrix permeability estimation. AAPG Bull..

[45] Kozeny J (1927). About capillary conduction of water in the soil (ascent, infiltration and application to irrigation): Vienna, Austria. Acad. Sci. Meeting Rep..

[46] Carman PC (1937). Fluid flow through granular beds. Trans. Inst. Chem. Eng..

